# Unique three-site compound heterozygous mutation in the WFS1 gene in Wolfram syndrome

**DOI:** 10.1186/s12902-021-00823-5

**Published:** 2021-08-17

**Authors:** Ziyu Ren, Jixiu Yi, Min Zhong, Yunting Wang, Qicong Liu, Xuan Wang, Dongfang Liu, Wei Ren

**Affiliations:** 1 Department of Endocrinology and Metabolism, The Second Affiliated Hospital of Chongqing Medical University, No. 74, Linjiang Road, Yuzhong District, Chongqing, 400010 China; 2 Department of Endocrinology and Metabolism, The Qianjiang Central Hospital, Chongqing, China; 3 Department of Neurological Disorders, Chongqing Medical University Affiliated Children’s Hospital, Chongqing, China; 4 Department of Endocrinology and Metabolism, The First Affiliated Hospital of Chongqing Medical University, No. 1, You-Yi Rd, Yu-zhong District, Chongqing, 400010 China

**Keywords:** Wolfram syndrome, Diabetes mellitus, WFS1, Compound heterozygous mutation

## Abstract

**Background:**

Wolfram syndrome (WFS) is a rare autosomal recessive genetic disease whose main cause is mutations in the WFS1 and CISD2 genes. Its characteristic clinical manifestations are diabetes insipidus, diabetes mellitus, optic atrophy and deafness.

**Methods:**

In this study, two patients from this particular family underwent complete routine biochemical and ophthalmic tests. Blood, urine, routine stool test, visual acuity (VA) examination, visual field assessment, funduscope, optical coherence tomography and periorbital magnetic resonance imaging (MRI) scans were performed for each patient to evaluate whether the nerve fiber layer around the optic nerve head was atrophied and next-generation sequencing of target genes was performed in two patients.

**Results:**

When the patients were diagnosed with Wolfram syndrome, their genetic analyses suggested unique three-site compound heterozygous mutations (c.2314C > T + c.2194C > T + c.2171C > T) in exon 8 of both patients’ chromosome 4. One mutation (c.2314C > T) was a novel mutation in the known reports of Wolfram syndrome. As a degenerative genetic disease, the types of gene mutations in the Chinese population are generally homozygous mutations at the unit point or compound heterozygous mutations at two nucleotide change sites. However, the two patients reported in this study are the first known cases of compound heterozygous mutations with three mutation sites coexisting on the WFS1 gene in China or even globally.

**Conclusions:**

This study expands the phenotypic spectrum of Wolfram syndrome and may reveal a novel mutation pattern of pathogenesis of Wolfram syndrome. The implications of this discovery are valuable in the clinical diagnosis, prognosis, and treatment of patients with WFS1.

## Background

WFS is an extremely rare autosomal recessive neurodegenerative disorder first identified by Wolfram and Wagener in 1938 [1]. The prevalence of WFS is generally extremely low, with a prevalence of 1 in 770,000 in the UK [2], but is relatively high in certain parts of Asia, such as Lebanon, with a prevalence of 1 in 68,000 [3]. The clinical manifestations of WFS are diverse and are mainly characterized by juvenile diabetes insipidus (DI), diabetes mellitus (DM), optic atrophy (OA) and deafness (D). The term “DIDMOAD” is also used to describe patients with WFS who have a variety of complications [4]. Subsequent studies have shown that only 51% of patients had signs of diabetes insipidus and deafness, and all four features are seen in only 13% of patients [4]. However, juvenile-onset diabetes mellitus and optic atrophy are always present and appear first. Therefore, juvenile diabetes and optic atrophy are the best diagnostic criteria for Wolfram syndrome [2, 5], and WFS is also classified as a special type of endocrine disease [6]. Because there is no specific treatment, the prognosis of WFS is extremely poor. Patients mainly have multiple systems of progressive neurodegeneration; initial symptoms include color vision and peripheral vision loss; mild hearing loss and diabetes insipidus gradually develop into optic atrophy, deafness and even ataxia, central nervous system dysfunction such as central apnea, and eventually death [7].

According to different pathogenic mechanisms, generalized WFS can be classified as Wolfram syndrome type 1 (WS1) and Wolfram syndrome type 2 (WS2) [2, 8]. WS1 is characterized by a pathogenic gene on chromosome 4. WS2 was first reported in 2000 in four consanguineous Jordanian families [8]. In contrast to the typical “DIDMOAD” symptoms of WF1, this new type of Wolfram syndrome presents unique clinical manifestations: peptic ulcer disease, bleeding tendency secondary to a platelet aggregation abnormality and absence of diabetes insipidus [9]. Follow-up studies have demonstrated that the pathogenesis of WS2 is due to a CISD2 mutation on chromosome 4q22–24 [8]. The WFS1 gene and the CISD2 gene participate in the separate regulation of two transmembrane proteins, “Wolframin” and “endoplasmic reticulum interferon-stimulating protein” (ERISP), on the endoplasmic reticulum [10, 11]. Wolframin is an endoplasmic reticulum transmembrane protein that contains nine transmembrane segments and is embedded in the membrane in an Ncyt/Clum topology [12] and assembles a high molecular weight complex of approximately 400 kDa in the membrane. It is widely overexpressed in the brain, pancreas, heart, and insulinoma beta cell lines. Its main function may be the homeostasis of Ca^2 +^ regulated transport in protein synthesis modification [13]. The ERISP encoded by CISD2 is a new type of transmembrane protein located on the endoplasmic reticulum membrane. ERISP has been demonstrated to have an effect similar to Wolframin in maintaining endoplasmic reticulum and cell homeostasis and Ca^2+^ transport [14].

## Methods

### Study design

In this study, we conducted a comprehensive clinical genetic investigation of a unique family case, systematically reviewed the clinical ophthalmology and endocrinology characteristics of two patients (brothers) in this family, and performed genetic analysis of their immediate family members in the previous two generations. We found that this case is different from the common single-site homozygous mutation or double-site compound heterozygous mutation type in that it is a rare three different site compound heterozygous mutation. We also identified 1 novel mutation in WFS1. Therefore, in this article, we report the first case of a three-site compound heterozygous mutation in China and the world.

### Study participants

#### Patient 1

The proband, an 18-year-old boy (eldest brother), was admitted to the hospital 9 years ago (9 years old) due to no obvious inducement of eye pain, haze, photophobia, tears with thirst, polydipsia, polyuria, and nocturnal enuresis. The initial diagnosis was “type 1 diabetes with double corneal ulcer”. After symptomatic treatment, such as anti-inflammatory and insulin therapy to control hyperglycemia, the patient was discharged from the hospital and received long-term insulin injection treatment outside the hospital. Two years ago (16 years old), due to the progressive decrease in binocular vision in the patient, he was diagnosed in our hospital as “left eye optic atrophy, left eye neurotrophic keratitis, and right eye neurotrophic corneal ulcer”. The diagnosis and treatment during this period are not quite clear. In recent years, the patient gradually felt hearing loss in the right ear, accompanied by increased urinary incontinence at night, so he was admitted to the hospital for further treatment.

#### Patient 2

Another case in this family was a 14-year-old boy (younger brother) who had symptoms similar to his elder brother’s thirst, polydipsia, polyuria, and nocturnal enuresis with blurred vision when he was 9 and was not treated. Three years ago (11 years old), his blood sugar increased, so oral “Chinese medicine” anti-hyperglycemic was administered for nearly 1 year. Two-plus years ago, there was a progressive increase in blurred vision, frequent urination, endless urination, and exacerbation of nocturnal enuresis. He was admitted to local hospital with “diabetic ketoacidosis,” and physicians performed the relevant examination (Table 1).

**Table 1:** Clinical characteristics of patients with Wolfram syndrome (DIDMOAD)

Case no.	Age	Sex	DI, age at diagnosis	DM, age at diagnosis	OA, age at diagnosis	HI, age at diagnosis	Other features, age at diagnosis	UCVA
**1**	18	M	No	Type 1, 9 years	Bilateral, 16 years	Left MF, 17 Years	Abnormal MRI of brain, uroschesis	OD: 0.04 OS: 0.1
**2**	14	M	No	Type 1, 9 years	Bilateral, 11 years	No	Abnormal MRI of brain, uroschesis	OD: 0.2 OS: 0.1

*DM* diabetes mellitus, *OA* optic atrophy, *DI* diabetes insipidus, *HI* hearing impairment, *MF* medium-frequency hearing impairment, *UCVA* uncorrected visual acuity, *EEG* Electroence phalography, *OD* right eye, *OS* left eye

### Laboratory analysis

Both patients underwent complete routine biochemical and ophthalmic tests Including blood, urine, stool routine test, VA examination, visual fields assessment (Carl Zeiss Meditec, Inc., Dublin, CA, United States), the funduscope, optical coherence tomography (Cirrus OCT 5000, Carl Zeiss Meditec, Inc., Dublin, CA, United States) and Periorbital MRI scans (philips Achieva 1.5 T, Netherlands) was performed for each patient to evaluate the nerve fiber layer around the optic nerve head is atrophied. Brain MRI was performed in 2 patients. The audiological, urological and psychiatric examinations results were recorded from the medical records.

### Genetic analysis

Extract the whole blood and DNA samples of the two patients and their Family members with the permission of the patient guardian. Genetic testing of two patients with next-generation sequences (NGS), A panel including 194 ophthalmology associated genes were sequenced by Illumina HiSeq 2000 (Illumina, Inc., San Diego, CA, United States) sequencing system. The average depth was 200x. Family members of the proband were validated by Sanger sequence.

## Results

Some of the examination results for both patients are as follows and are presented in Table 2. All the clinical details and images of the patients mentioned later were obtained with the consent of their parents. Patients and participants provided their written informed consent to participate in this study.

**Table 2:** Routine laboratory testing of patients with Wolfram syndrome

Case no.		Endocrine routine			TC		Routine urine test	
	HbA1c % (mmol/mol)	FBG (mmol/L)	C-P (nmol/L)	Cholesterol (mmol/L)	Triglyceride (mmol/L)	UGLU	Urine ketone body	Urine specific gravity
**1**	12.9	15.01	0.07	5.22	1.09	++++	Positive	1.029
**2**	8.4	10.79	0.05	4.71	0.87	+++	Positive	1021

*HBA1c* glycated hemoglobin, *FBG* fasting blood glucose, *C-P* c-peptide, *TC* triglyceride, *UGLU* urine glucose

### Patient 1 (eldest brother, proband)

Fasting blood glucose was 15.01 mmol/L, fasting c-peptide was 0.07 nmol/L, and glycosylated hemoglobin (HbA1c/Ghba1c) was 12.8%.

### Patient 2 (younger brother)

Fasting blood glucose was 10.79 mmol/L, fasting c-peptide was 0.33 nmol/L, HbA1c/Ghba1c was 8.4%, and he was urine sugar and urine ketone body positive.

**Fig. 1:**
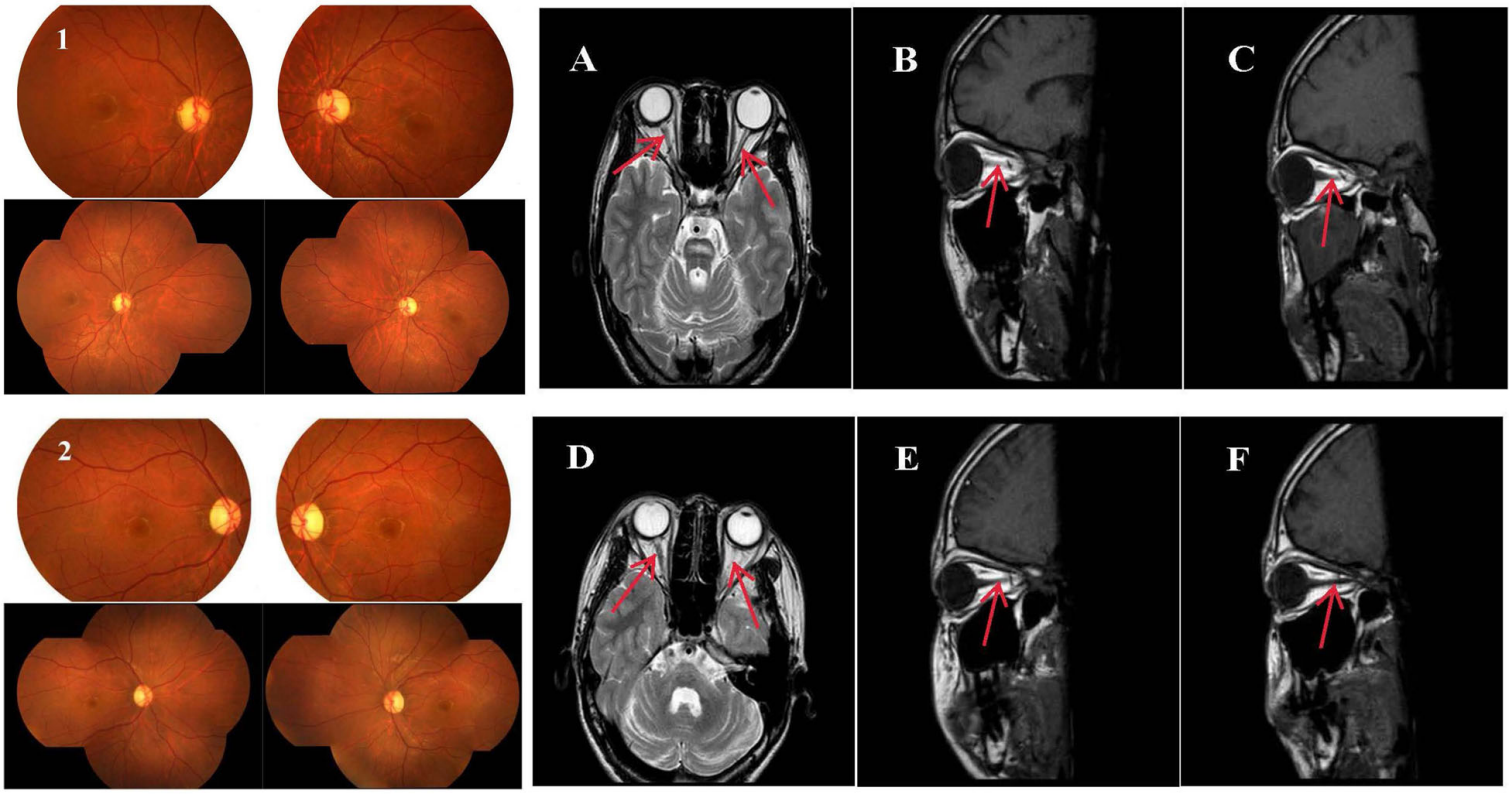
Fundus photography and periorbital magnetic resonance images of two patients. (1) Shows the fundoscopic results of patient 1, and (2) shows the fundoscopic results of patient 2. Both figures show optic disc diffused pallid bilaterally without diabetic retinopathy. The two patients’ optic nerves were pale, and the bilateral optic nerves were thinner. **A-C** Bilateral optic nerve atrophy from the coronal and sagittal positions in patient 1. **D-F** Bilateral optic nerve atrophy from the coronal and sagittal positions in patient 2. Red arrows show the atrophic optic nerves of the two patients

**Fig. 2:**
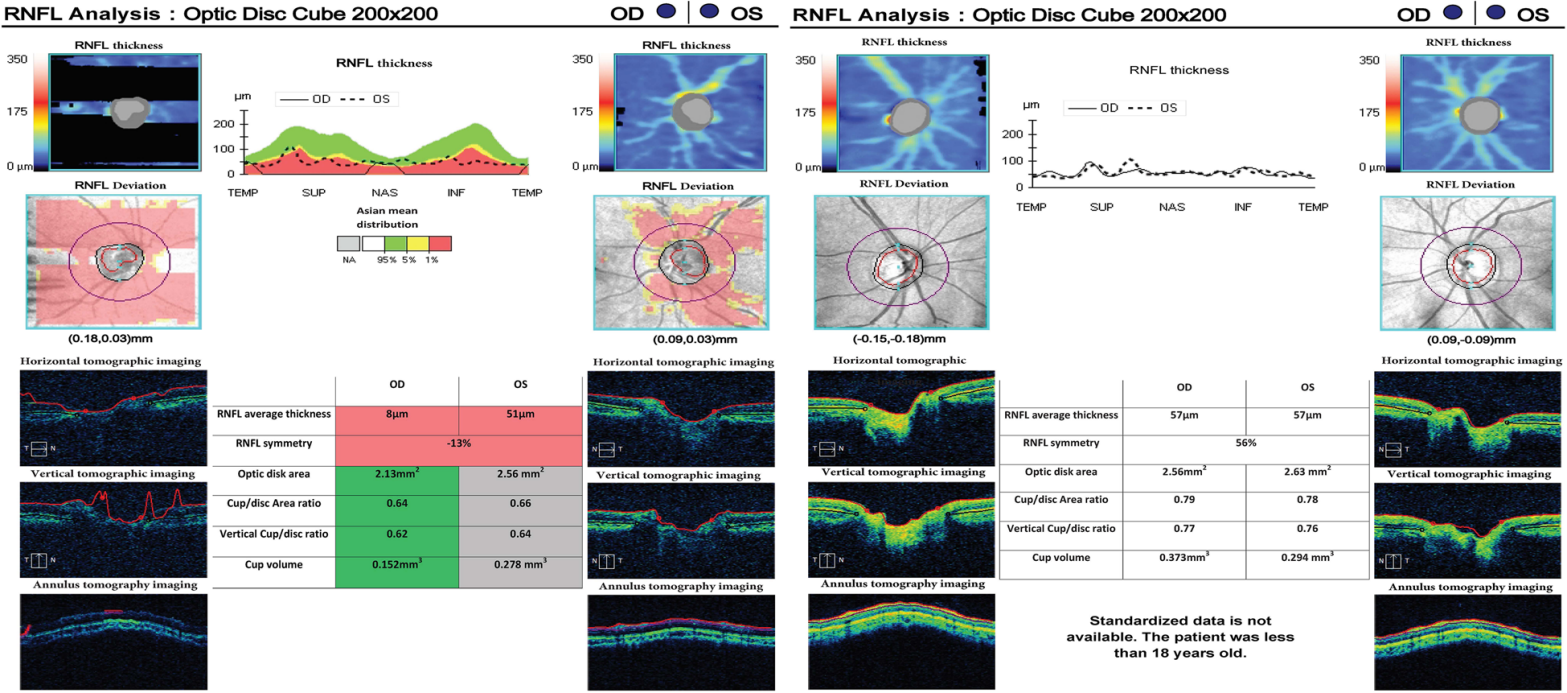
Optic coherence tomography of two patients with WFS1 mutation. Optic coherence tomography (OCT) shows that peripapillary retinal nerve fiber layer (RNFL) thickness significantly decreases. The left graph shows the thickness of the retinal nerve fiber layer in both eyes of patient 1. The right graph shows retinal thickness in both eyes of patient 2. The red part represents a decrease in thickness less than 1% outside normal, and the green part indicates a decrease within the normal limit. Patient 2 represented in the right figure cannot be measured with standardized data because he is under 18 years of age. RNFL: retinal nerve fiber layer; OD: right eye; OS: left eye

Both patients had typical symptoms of thirst, polydipsia, polyuria and weight loss, so they were diagnosed with type 1 diabetes mellitus (DM1). The patient’s cholesterol, triglyceride, HDL cholesterol, LDL cholesterol and thyroid function were all normal. Optic coherence tomography (OCT) was performed for each patient to evaluate retinal nerve fiber layer (RNFL) thickness. Ophthalmic fundoscopy and periorbital MRI suggested that the ratio of the optic cup to the optic disc was unclear, the optic nerve was pale, and the bilateral optic nerve was thinner, which are consistent with the diagnosis of OA (Figs. 1 and 2). DM1 combined with OA meets the basic diagnostic criteria of WFS. The pure tone listening test (PTA) revealed that patient 2 had moderate left conductive deafness. Brain MRI scans indicated that brainstem and cerebellar vermis atrophy were accompanied by brainstem lamellar abnormal signal shadows in the two patients. No obvious abnormality was found by chest radiograph and abdominal B-ultrasound.

#### Genetic analysis

**Fig. 3:**
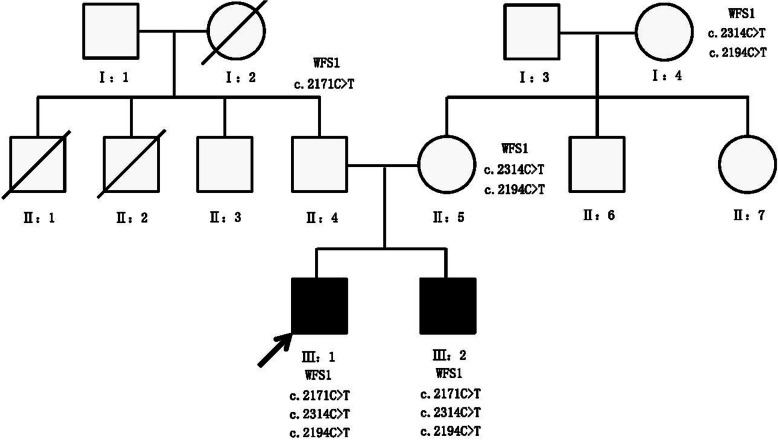
Pedigrees of this WFS family. Black squares: affected males; white squares: unaffected males; white circles: unaffected females; arrow: the proband

**Fig. 4:**
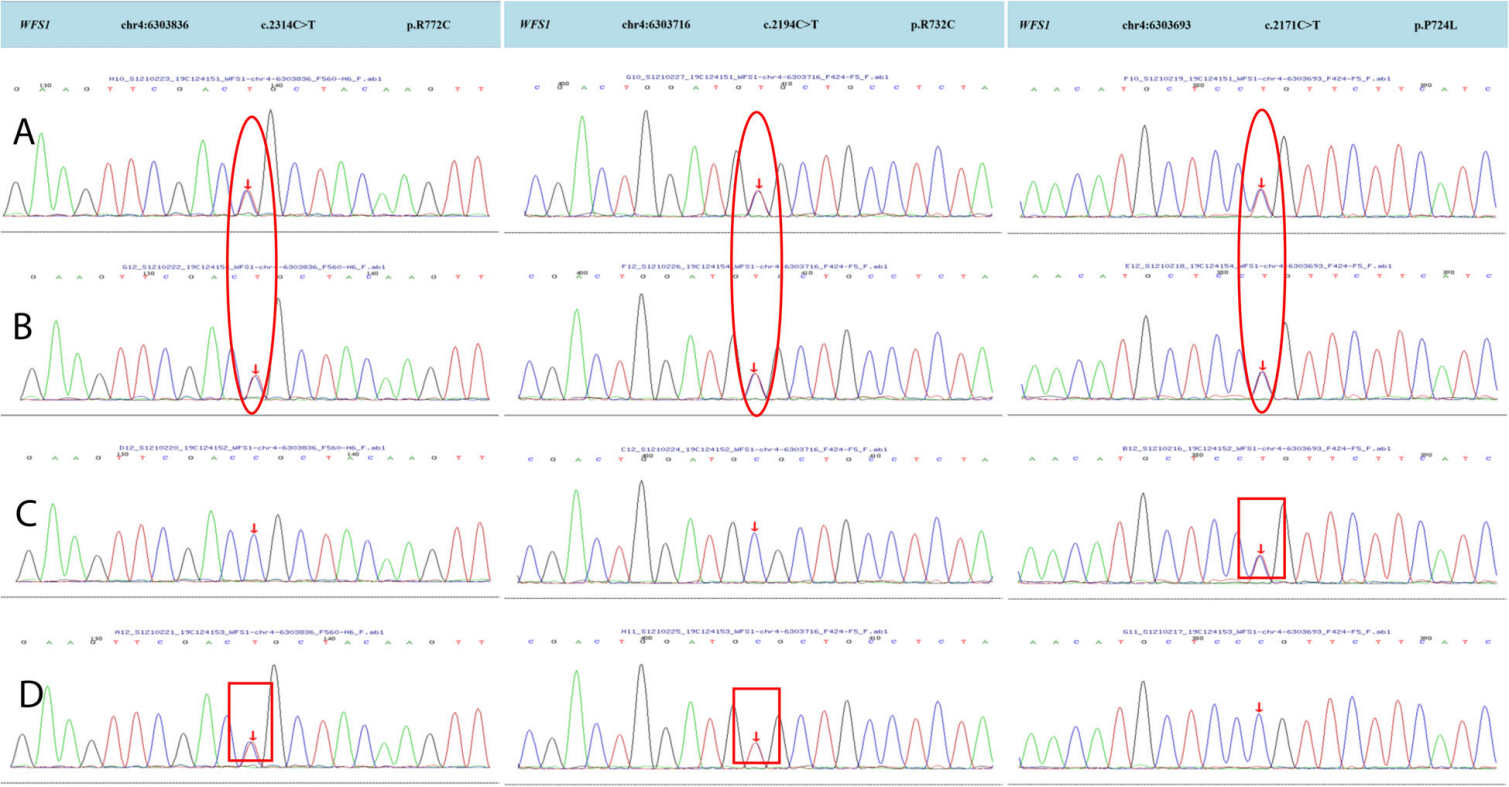
High-throughput sequencing results of WFS1 in both patients and Sanger sequencing results of WFS1 in their parents. **A**, **B**, **C**, **D** The genetic sequencing results of patient 1, patient 2, father, and mother, respectively. Patient 1 and patient 2 had all three heterozygous mutations in exon 8 of the WFS1 gene. The father and mother of the patients had heterozygous mutations (c.2171C > T (p.P724L) and c.2314C > T (p.R772C) + c.2194C > T (p.R732C), respectively. The red circle presents heterozygous mutations in the two patients. The red box presents heterozygous mutations in their parents

**Fig. 5:**
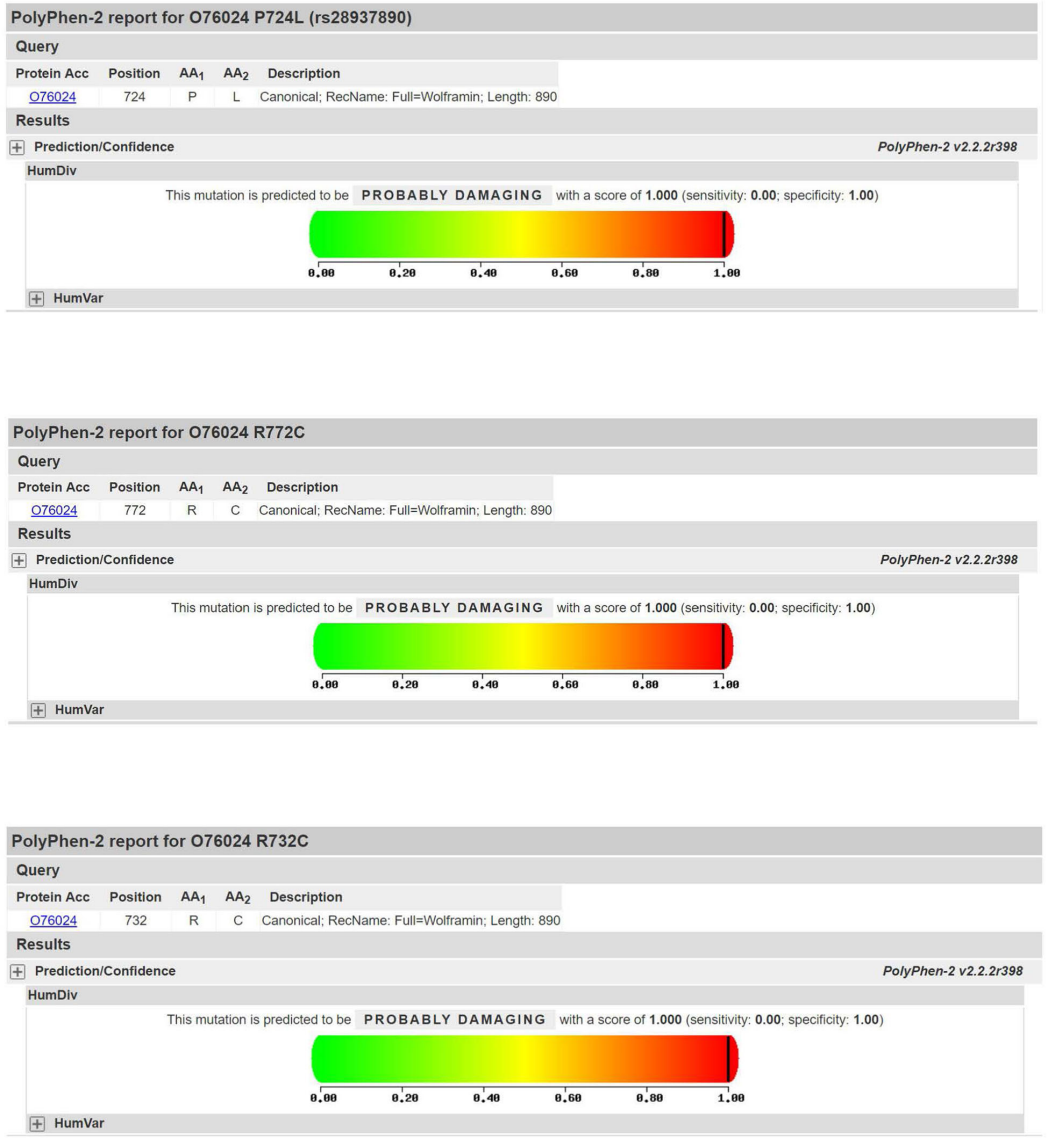
Protein function prediction for three mutant sites (c.2171C > T (p.P724L); c.2314C > T (p.R772C); c.2194C > T (p.R732C)) of Polyphen2

Both patients had mutations in the WFS1 gene, and both had unique three-site compound heterozygous mutations. No other gene mutations or mitochondrial genomic mutations were detected. Pedigrees of this WFS family in our study are shown in Fig. 3. The mutation sites of these two patients were located in exon 8 of the WFS 1 gene c.2314C > T (p.R772C) + c.2194C > T (p.R732C) + c.2171C > T (p.P724L) (Fig. 4). Both of these mutations were inherited from their maternal grandmother (Table 3). The c.2194C > T (p.R732C) and c.2314C > T (p.R772C) loci were searched using PP3 bioinformatics protein function prediction software, including SIFT, PolyPhen_2, MutationTaster, GERP ++, REVEL, all of which indicate harmfulness (Fig. 5).

**Table 3:** Sanger sequencing reveals WFS1 gene mutations and clinical manifestations in the patients’ maternal grandparents

Test gene	Detection location	Detection method	Nucleotide changes	Subject	Clinical manifestation	Results
**WFS1**	chr4–6,303,836	Sanger sequencing	c.2314C > T	maternal grandfather	negative	**No variation**
				maternal grandmother	negative	**Heterozygous mutations**
**WFS1**	chr4–6,303,716	Sanger sequencing	c.2194C > T	maternal grandfather	negative	**No variation**
				maternal grandmother	negative	**Heterozygous mutations**

#### Literature review

We systematically identified all potentially relevant articles from PubMed and Web of Science, Search terms about “Wolfram syndrome” and “WFS1” and “CISD2” and “gene mutation sites”, were used in various combinations and permutations across the databases.There were a total of 26 papers. We selected case reports of only two types of wolfram syndrome, including cases with mutations at different nucleotide loci on two pathogenic genes between 2011 and 2019. After screening and sorting, We eliminated wolfram syndrome-related mechanistic studies and reviews, excluded duplicate loci reported in the literature, and finally selected 12 articles [11, 14–24]. All 12 articles reported reports of disease caused by mutations at different nucleotide sites on the WFS1 and CSDI2 genes. (Table 4).

**Table 4:** Clinical mutated nucleotide site and patterns of patients in WFS1 and CISD2 genes of Wolfram syndrome

GENE	Population	Nucleotide changes	Exon	Zygosity	References
WFS1	Polish	c.1232 V > delGCTG	Exon8	Homozygous	
WFS1	Polish	c. 1943G > A c. 2336 T > G	Exon8	compound heterozygote	[15]
WFS1	Polish	c. 1330C > G	Exon8	Homozygous	
WFS1	Iranian	c.376G > A	Exon8	homozygous	[16]
WFS1	Iranian	c.1672C > T	Exon10	homozygous	[17]
WFS1	Iranian	c.330C > A	Exon4	Homozygote	[22]
WFS1	Turkish	c.1832_11847del16 c.1672C > T	Exon 8	Compound heterozygote	
	Turkish	c.1867delA c.1943G > A	Exon 8	Compound heterozygote	[18]
	Turkish	c.376G > A	Exon 4	Homozygote	
WFS1	Chinese	c.1760G > A	Exon 8	Homozygote	[19]
WFS1	Japanese	p. N325_I328del		heterozygote Homozygote	[21]
CISD2	Chinese	c.272_273del	Exon 2	Homozygote	[20]
CISD2	Moroccan	c.215A > G	Exon 2	Homozygote	[14]
CISD2	Italian	c.103 + 1G > A	Intron 1	Homozygote	[23]
CISD2	Caucasian	Intragenic deletion	Exon 2	Homozygote	[24]
CISD2	Jordanian	c.109G > C	Exon 2	Homozygote	[11]
**WFS1**	**Chinese**	**c.2314C > T** **c.2194C > T** **c.2171C > T**	**Exon8**	**Compound heterozygote**	**This study**

All papers are case reports on single nucleotide polymorphisms on the WFS1 and CISD2 genes in wolfram syndrome. According to available literature, patients with wolfram syndrome are mostly female (21 female, 9 male), all aged between 9 and 24 years old. The age of onset tends to be younger. Wolfram patients tend to show both severe juvenile diabetes and optic nerve atrophy to varying degrees at a very early stage. The disease is often diagnosed by genetic testing several years after the first symptoms of DOMIO have progressively worsened. Among these 26 papers, five patients had wolfram syndrome caused by mutations in the CISD2 gene, while the rest had disease caused by mutations at different nucleotide sites in exons 4, 5 and 8 of the WFS1 gene. Wolfram syndrome caused by the CISD2 gene mutation exhibits very different clinical features, characterized by peptic ulcer disease, bleeding tendencies secondary to abnormal platelet aggregation and absence of enuresis. Their survival time was also longer compared to WFS1.

## Discussion

In this study, we evaluated a unique family case. In addition to the rare three-site compound mutation type, c.2314C > T is the first reported new mutant site. Both patients had diabetes as the first symptom at their early stage accompanied by at least one of the characteristics of WFS, which is consistent with the diagnostic criteria of WFS [2]. During the progression of the disease, the ophthalmic symptoms of these two patients appeared earlier and gradually worsened. Various ophthalmological examinations show abnormalities (severe vision loss, decreased color vision, narrow field of vision, abnormal visual evoked potential, etc.) that need to be distinguished from diabetic retinopathy [25].

Recently, studies have confirmed that the pathogenesis of WFS is closely related to mutations in two genes, WFS1 and CISD2. Where WFS1 regulates wolframin, a functional protein encoded in the endoplasmic reticulum (ER), which is involved in posttranslational modification, folding, and assembly of new synthetic proteins, such as insulin, calcium storage, REDOX regulation, steroid synthesis, and apoptosis [5]. Changes in ER function lead to accumulation of misfolded proteins and activation of the unfolded protein response (UPR), a state known as “ER stress” [26]. However, WFS1 plays a key role in ER stress, and recessive or dominant mutations in WFS1 consistently lead to neuronal and/or endocrine dysfunctions [27, 28]. According to existing reports, the mutation sites in the two genes are diverse, but all of them are homozygous mutations and double-site compound heterozygous mutations (Table 4). However, the pathogenic pattern of this family is a unique “three-site” compound heterozygous mutation. Mutations in the WFS1 gene in three generations of the patient’s family can be clearly seen in Fig. 4. The patients’ family pedigree shows that heterozygous mutations at two nucleotide change sites (c.2194C > T, c.2314C > T) in the WSF1 gene have been present since the patients’ grandmother (older relatives were unable to provide blood samples due to age issues). The prediction of protein function also suggested that the muations in the encoded at all three sites of the protein were harmful. The conventional wisdom is that this type of mutation should be pathogenic, but interestingly, the two-site compound heterozygous mutation in the patient’s mother and grandmother did not cause the disease. Through Sanger sequence verification, the heterozygous mutation c.2171C > T(p.P724L) in the WSF1 gene was identified in the father, which was not causing disease. Unfortunately, two young male patients in this family underwent genetic selection and had three different nucleotide mutations inherited from their parents’ chromosomes at the same time. It is likely that this unprecedented “three-site” compound heterozygous mutation caused the onset of WFS in the two young men.

It is worth noting that this type of mutation pattern in WFS1 has never been reported in the literature. The nonpathogenic single-site heterozygous mutation and the compound heterozygous mutations in the WFS1 gene on chromosome 4 of the parents were brought together on the same pair of chromosomes through genetic recombination and eventually caused disease. This case likely reveals a new WFS pathogenic pattern. We can boldly assume that the pathogenic ability of the heterozygous mutation site in the WFS1 gene may have a “cumulative effect”. Mutational sites may acquire greater pathogenicity through multiple generations of genetic accumulation and eventually lead to disease. This hypothesis was confirmed in our study, in which two patients developed disease due to the simultaneous acquisition of both parental heterozygous mutation sites. As WFS is an extremely complex neurodegenerative genetic disease, its specific pathogenesis is not clear. At this stage of our study, there is a lack of subsequent functional studies of genes and proteins. The mechanism of the three-site mutation pattern also needs to be confirmed by more case evidence and subsequent multidisciplinary and multiteam cooperation research. This is also the direction of this team’s follow-up research.

The mother of the two patients in this case is currently pregnant with her third child and has been pregnant for approximately 10 weeks. It is precisely because of the unique pathogenic pattern and genetic mutation site of her family that we recommend close monitoring of the child’s growth after birth, as well as appropriate screening to ensure quality of life. This case also suggests that endocrinologists need to be vigilant in clinical work. The possibility of WFS should be considered in young DM patients with early vision loss. They should also be advised to obtain a thorough prenatal test when their family members are ready to become pregnant [29]. WFS as a special category of endocrine diseases [ICD-11] that should receive more attention. We also strongly recommend that patients with typical DM1 combined with ophthalmic symptoms undergo genetic testing to determine the disease type for subsequent treatment [30].

## Conclusions

Patients with juvenile onset diabetes with optic nerve atrophy should be alerted to the possibility of WFS and should undergo aggressive genetic analysis. Three-site compound heterozygous mutation may be a potential novel pathogenesis of WFS that deserves further in-depth study.

## Data Availability

The datasets generated and analysed during the current study are available in the [National Center for Biotechnology Information] repository, [https://www.ncbi.nlm.nih.gov/gene/7466]. The datasets generated and analysed during the current study are available in the [Polymorphism Phenotyping v2] repository, [http://genetics.bwh.harvard.edu/pph2/].

## Abbreviations

WFS: Wolfram Syndrome
VA: Visual Acuity
MRI: Magnetic Resonance Imaging
DI: Diabetes Insipidus
DM: Diabetes Mellitus
OA: Optic Atrophy
ERISP: Endoplasmic Reticulum Interferon-stimulating Protein
NGS: Next-generation Sequences
HbA1c/Ghba1c: Glycosylated Hemoglobin
DM1: Type 1 Diabetes Mellitus
OCT: Optic coherence tomography
RNFL: Retinal nerve fiber layer
PTA: Pure tone listening test
UPR: Unfolded protein response
OD: Right eye
OS: Left eye
HI: Hearing impairment
MF: Medium-frequency hearing impairment
UCVA: Uncorrected visual acuity
EEG: Electroence phalography
FBG: Fasting blood glucose
C-P: C-peptide
TC: Triglyceride
UGLU: Urine glucose
